# Automating Fault Test Cases Generation and Execution for Automotive Safety Validation via NLP and HIL Simulation

**DOI:** 10.3390/s24103145

**Published:** 2024-05-15

**Authors:** Ayman Amyan, Mohammad Abboush, Christoph Knieke, Andreas Rausch

**Affiliations:** Institute for Software and Systems Engineering, Technical University of Clausthal, 38678 Clausthal-Zellerfeld, Germany; ayman.amyan@tu-clausthal.de (A.A.); christoph.knieke@tu-clausthal.de (C.K.); andreas.rausch@tu-clausthal.de (A.R.)

**Keywords:** automotive software systems, fault injection (FI), hardware-in-the-loop (HiL), real time, ISO 26262, natural language processing (NLP), bidirectional encoder representations from transformers (BERT), Word2Vec

## Abstract

The complexity and the criticality of automotive electronic implanted systems are steadily advancing and that is especially the case for automotive software development. ISO 26262 describes requirements for the development process to confirm the safety of such complex systems. Among these requirements, fault injection is a reliable technique to assess the effectiveness of safety mechanisms and verify the correct implementation of the safety requirements. However, the method of injecting the fault in the system under test in many cases is still manual and depends on an expert, requiring a high level of knowledge of the system. In complex systems, it consumes time, is difficult to execute, and takes effort, because the testers limit the fault injection experiments and inject the minimum number of possible test cases. Fault injection enables testers to identify and address potential issues with a system under test before they become actual problems. In the automotive industry, failures can have serious hazards. In these systems, it is essential to ensure that the system can operate safely even in the presence of faults. We propose an approach using natural language processing (NLP) technologies to automatically derive the fault test cases from the functional safety requirements (FSRs) and execute them automatically by hardware-in-the-loop (HIL) in real time according to the black-box concept and the ISO 26262 standard. The approach demonstrates effectiveness in automatically identifying fault injection locations and conditions, simplifying the testing process, and providing a scalable solution for various safety-critical systems.

## 1. Introduction

In the context of maintaining operational safety in all scenarios, an important obstacle is the ever-increasing complexity of automotive software systems. This complexity is fueled by the never-ending search for new features and improved functionality, particularly in the field of autonomous and semi-autonomous vehicles [1]. Because real-world driving environments are dynamic and unpredictable, it becomes increasingly difficult to anticipate and test against every possible fault condition [2]. To verify the resilience of these intricate systems, the ISO 26262 Functional Safety Standard [3], recommends the methodical application of fault injection techniques [4].

The motivation for exploring the evolution of vehicles with advanced driver-assistance systems (ADAS), autonomous driving capabilities, and Internet of Things (IoT) integrations lies in the increasing complexity of their operating systems [5]. Fault injection tests the reliability of these interconnected systems to ensure that a fault in one component does not lead to a comprehensive system breakdown [6]. The essence of fault injection lies in its proactive nature; therefore, engineers can identify and address vulnerabilities before they manifest in real-world conditions [7]. Moreover, fault injection is instrumental in complying with automotive safety standards, such as ISO 26262 [6].

The problem with fault injection as a methodology for improving automotive safety systems includes several challenges:

- The complexity of manually simulating realistic fault conditions in safety-critical systems increases the potential for human error and oversight in critical testing phases.
- Manual injection processes and expertise dependency. Complexity and time consumption due to the integration of many sensors and actuators with control units (ECUs) [8].
- Limited test case execution testers, given the time and effort constraints, limit fault injection cases by ignoring less obvious scenarios, which can potentially be critical [9].
- Difficulty in simulating realistic faults because of the complex conditions during the experiment. Automating the fault injection process presents both a challenge and an opportunity.
- Automotive systems are subject to strict regulatory standards such as ISO 26262. Ensuring that fault injection methodologies comply with these standards and that they do not inadvertently compromise the system.

The proposed solution in this paper is a pioneering approach for automatically generating fault test cases that are directly based on functional safety requirements using natural language processing (NLP), especially Bidirectional Encoder Representations from Transformers (BERT) [10], and Word2Vec [11], setting a new standard for precision in automotive safety validations. With the help of dSPACE’s toolkit and a fault types library, it is possible to conduct an entirely automated, end-to-end testing process that starts from a requirement analysis to the generation of experiment reports [12,13]. The fault is also injected in real time [14] to ensure a true representation of the real-life fault injection experience. Additionally, by using a black-box testing methodology [15], this approach makes sure to avoid any potential biases or artifacts resulting from internal manipulation and maintains the integrity of the system being tested.

This paper contributes to the field by introducing several major innovations:

1. The automatic generation of fault test cases directly from functional safety requirements, utilizing advanced natural language processing techniques, reduces manual effort, accelerates testing cycles, and enhances adaptability in testing protocols as system requirements evolve.
2. The automatic execution of these generated fault test cases, adhering to the principles of the black-box testing concept to guarantee unbiased results.
3. The incorporation of real-time fault injection capabilities to simulate faults in complex systems enhances the realism and applicability of tests.
4. Single- and multi-fault injection techniques to evaluate system resilience under varied conditions and scenarios.
5. Comprehensive coverage of all expected communication faults derived from the functional safety requirements, ensuring an in-depth testing process.
6. The flexible methodology offers extensive applicability beyond automotive safety, potentially transforming practices in the aerospace and industrial automation sectors.
7. Automated testing ensures consistent test execution, reducing human error and increasing the accuracy and reproducibility of results. Efficiently handles increased system complexity irrespective of the architectural scale.

The remainder of this paper is structured as follows: Section 2 delves into the fundamentals of fault injection and the theoretical underpinnings of BERT. Section 3 outlines the methodology, including the process of automatic generation and execution of fault test cases. Section 4 provides a comprehensive case study, illustrating the practical application of our methodology in real-world scenarios. Section 5 offers a thorough analysis of the case study’s outcomes, focusing on the effectiveness and precision of the proposed approach. The paper concludes with Section 6, where we summarize our key insights, discuss the implications for enhancing automotive software systems’ safety validations, and suggest avenues for future research.

## 2. Background and Overview

This section provides a foundational overview of fault injection, NLP technology, and the HIL simulation platform. We explore fault injection’s role in verifying system robustness against failures, guided by ISO 26262 standards, and its integration into the development lifecycle for ensuring automotive system reliability. Simultaneously, we introduce BERT, a breakthrough in natural language processing, demonstrating its potential to automate fault test case generation with its deep learning capabilities and additionally presenting the most important benefits of the HIL simulation platform.

### 2.1. Fault Injection Approach

Fault injection (FI) is an approach to assess the robustness of fault-tolerant systems [12]. By intentionally injecting faults into the system [16]. This methodology is of great value in evaluating the suitability of fault detection and recovery mechanisms. In addition, it helps to identify and mitigate potential risks [17].

Fault injection is an important multi-objective technique that mainly focuses on testing the robustness of the system for random faults rather than validating the functionality [9,18], fault recovery [9,12], and ensures that safety requirements are not violated [17].

The ISO 26262 standard’s recommendations state that the deliberate selection of fault injection locations in automotive software development is essential to system integrity and reliability. To verify discrete functionality, the standard suggests injecting random faults into individual software modules or components during unit testing. To confirm the efficacy of safety mechanisms in the integrated system, integration testing entails purposefully inserting defects into both software and hardware components [19].

Fault injects according to the following attributes:

- Fault Location (Where to Inject): To enable realistic testing scenarios, any sensor or actuator inside the vehicle can be a potential site for these injections.
- Fault Type (What to inject): This paper includes a wide variety of fault types. These faults fall into various categories [20], i.e., value-based faults, which comprise **Stuck-at**, **Offset**, and **Gain** faults; faults causing disturbance to signals including **Drift**, **Spike**, and **Noise** faults, including **Hard-over** Faults under the Threshold and Extreme Value Faults with the Maximum Threshold; and **Packet loss** and **delay** faults fall under the fourth category of network and communication faults.
- Fault Time (When to Inject): There are two types of fault injection timing in fault testing: event-dependent and time-dependent. **Time-dependent fault injection** involves the introduction of faults after a predefined experiment duration, which can be set by the user or ascertained using a probability distribution. In contrast, **event-dependent fault injection** involves the activation of faults in response to events that transpire during system execution. For example, a fault arises when a vehicle reaches a speed of more than 100 km per hour. Both time-based and event-based fault injection scenarios can be handled by our method with effectiveness.

A fault injection environment is one in which several parts collaborate to assess and improve system reliability. These include according to [12] the following: (1) Fault Injector: It causes faults to be introduced into the system. (2) Fault Library: It includes a variety of fault kinds and traits. (3) Workload Generator: It generates system workload inputs. (4) Workload Library: It offers a selection of workloads that are already set. (5) Controller: It oversees and plans the experiment involving fault injection. (6) The monitor starts gathering data and monitors system execution. (7) Data Collector: Throughout the experiment, it gathers data in real-time. (8) Data Analyst: They handle and evaluate the information gathered.

Three general types of fault injection strategies can be distinguished: (1) data fault injection [21,22]; (2) interface fault injection [23,24]; and (3) modifications to the code [25,26]. We are using the **communication data fault injection** in this project, by manipulating the signal according to Equation [20]:

(1) $$f\left(t\right)=d_{v}h\left(t\right)+O_{v}$$

where f(t) is the faulty or manipulated signal value, $d_{v}$ represents the gain value, h(t) is the healthy or standard signal value, and $O_{v}$ represents the offset/bias value. We will be able to carefully watch and examine how the system responds to anomalies in the data thanks to this technique.

Fault injection techniques improve security requirements, assess the efficacy of recovery mechanisms, and simulate intentional or random errors: (1) hardware-based fault injection [16]; (2) software-based fault injection [12]; (3) simulation-based fault injection [12,18]; and (4) emulation-based fault injection [13,16].

We have decided to use **software-based fault injection** for this project. This method is preferred because of its adaptability, thorough fault injection capabilities, and capacity to change the system state adaptively without requiring extra hardware. This approach provides an excellent balance of thoroughness, adaptability, and cost-efficiency, which is in line with our project requirements.

Several noteworthy tools and approaches stand out in the field of fault injection: basic software-implemented fault injection models are implemented for automated testing by *FIAT* [27]. Error models for distributed systems are the area of expertise for *DOCTOR* and *ORCHESTRA* [28]. Stress- and path-based injection techniques are used by *FTAPE* [29]. The main goal of *SymPLFIED* is to use symbolic execution for data error injection [30]. Operation system debugging techniques are used by *FERRARI* to perform fault injection [31]. *Xception* introduces faults through CPU debugging mechanisms [32].

### 2.2. Integrating Fault Injection into the Development Life Cycle according to ISO 26262

The present state of automotive software fault injection testing tools is limited, and ISO 26262 [3], despite its comprehensive approach, does not specifically specify the locations and timing of fault injection. According to the standard, fault injection testing should be used during the whole development process and the V-Model should be used when developing automotive systems. System development, hardware, and software phases are all included in this [33].

With an emphasis on continuous operation, reliability in system modules is defined as the likelihood of constant, failure-free performance over time under specific circumstances. This is not the safety case, which is the system’s ability to function or stop working without endangering users or the environment [16]. The treatment of safety as a system characteristic raises questions about its applicability and presents challenges in complex dynamic systems, whereas reliability is typically viewed as a module attribute under defined conditions [34].

Safety and reliability are guaranteed by ISO 26262 standards. The continuous safety lifecycle in the automotive industry is covered in five of the twelve parts that make up the ISO 26262 standard. These parts are [35] Part 3, Concept Phase [36,37]; Part 4, System Safety Integration and System-Level Product Development; Part 5, Hardware Safety Focus; Part 6, Development of software safety and software-level product; Part 7, Production and Operation; and Part 9, Automotive Safety Integrity Level (ASIL)-oriented and safety-oriented analyses [3].

**Figure 1:**
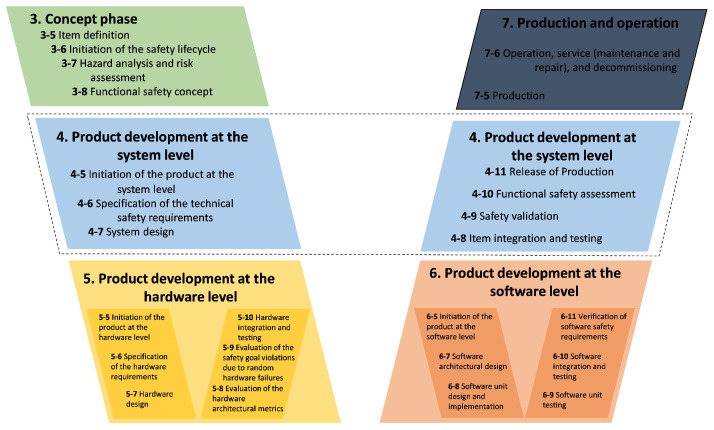
ISO 26262 Functional Safety Life Cycle.

For automotive systems to maintain product safety, ISO 26262, in all its components, requires a thorough approach at every stage, which is implemented by the V-Model and guarantees both safety and reliability, as shown in Figure 1.

### 2.3. Natural Language Processing (NLP)

Natural Language Processing (NLP) merges linguistics, artificial intelligence, and computer science to make interactions between computers and human language [38]. It includes tasks ranging from simple sentence segmentation to complex semantic annotation and opinion mining [39,40]. Essential NLP tasks include speech recognition, part of speech tagging, word sense disambiguation, named entity recognition, co-reference resolution, sentiment analysis, and natural language generation [38,41,42]. These processes enable computers to understand and generate language, accommodating nuances like accents and colloquialisms.

NLP utilizes machine learning techniques such as supervised learning on task-specific datasets, which are computationally intensive [43]. Techniques like word embeddings (e.g., GloVe, Word2Vec) transform text into numerical vectors to understand semantic similarities [40,43]. Advanced models like CNNs and RNNs further enhance NLP systems, allowing them to learn from large data and improve the accuracy in processing text and voice data.

#### Bidirectional Encoder Representations from Transformers (BERT)

A game-changing development in the field of natural language processing (NLP) is BERT (Bidirectional Encoder Representations from Transformers) [10]. Because of its versatility and efficiency, it has advanced in eleven different NLP tasks [44].

BERT’s architectural foundation is the Transformer model [45,46], which is renowned for its attention mechanisms that painstakingly comprehend a sentence’s word context [47,48]. The Transformer uses a bidirectional approach, and BERT can capture the context of each word [10].

During BERT’s pre-training phase, BERT can learn basic language structures and patterns as well as syntax, grammar, and contextual subtleties [49,50]. Two important methods employed here are Next Sentence Prediction (NSP) [51,52] and the Masked Language Model (MLM) [53,54,55].

During the fine-tuning phase [10], BERT’s general linguistic expertise is refined for use. Additional output layers are added to the model, each specifically tailored for NLP tasks. This phase is devoted to optimizing BERT’s broad language interpretation for tasks, necessitating smaller, more focused datasets [56,57].

### 2.4. Hardware-in-the-Loop (HIL)

A technique that synergistically combines virtual and physical prototyping is called hardware-in-the-loop (HIL) simulation. By combining physical copies of some subsystems with a closed-loop virtual simulation of the other subsystems, it emulates a system. When a system’s design develops and physical prototyping becomes more necessary, this method is especially helpful [58,59].

For the development of complex systems like powertrain controllers, automotive safety systems, unmanned underwater vehicles, and defense systems, HIL simulation is essential [60]. Cost-effectiveness, quick prototyping, high fidelity, simulation speed, repeatability, non-destructive nature, wide range, safety, and the ability to support concurrent systems engineering are just a few of its benefits [61,62].

## 3. Related Works

In this section, we summarize pioneering methodologies in fault injection testing and automotive system safety validations. Highlighting the progression from manual to automated testing techniques, this section contextualizes the advent of combining property-based testing with fault injection, leveraging machine learning for system model reverse-engineering and employing simulation for fault injection. These contributions set a backdrop for our proposed solution in Section 1, where we advance this domain by harnessing NLP technologies for the automated generation and execution of fault test cases, aiming to enhance the precision and efficiency of validating automotive software systems.

In [63], the method for creating fault test cases combines property-based testing (PBT) and fault injection (FI), in which PBT creates tests automatically based on system properties and FI expedites the occurrence of faults for the assessment. Functional requirements are the source of fault cases, which incorporate FI into PBT models. The methodology uses automated fault generation and injection with tools such as *QuickCheck* and *FaultCheck* and supports both single and multi-fault injections. The testing strategy combines black-box and white-box techniques, emphasizing both internal workings and outward behaviors.

Svenningsson et al. describe an approach for fault test case derivation and execution in MATLAB/Simulink models in “The *MODIFI* tool”. To test the system’s resilience to faults and monitor compliance with safety regulations, automated fault injection is used. While the tool can handle multiple faults, it initially focuses on single faults. Using a combination of black-box and white-box testing techniques, this approach automates the generation and execution of fault tests. Real-time fault injection, however, is not used in this approach [64].

Reiter et al. offer a process for deriving fault test cases using both Error Effect Simulations (EES) and Component Fault Trees (CFTs). This method supports both single and multi-fault injections and guarantees that the fault test cases meet functional safety standards. CFTs and EES are used to automate the creation and execution of fault test cases. Focusing on internal logic and external effects, the testing strategy blends elements of white-box and black-box testing. This paper [65] does not address real-time fault injection.

In [66], a machine learning technique for reverse-engineering system models for the generation of fault test cases is presented. This method employs model checking and automatically generates test cases based on defined safety requirements. Using learning-based techniques and hardware emulation, the generation and execution of fault test cases are automated. It focuses on external system behaviors and applies a black-box testing methodology.

Saraoglu et al. use MATLAB Simulink to develop a methodology for simulation-based fault injection in autonomous driving systems. Through component faults, this method evaluates the system’s compliance with safety objectives. The fault injection is intended for single or multiple fault injections. With the simulation framework, which combines aspects of white-box and black-box testing techniques, fault test cases are automatically generated and executed [67].

In [68], a real-time fault injection framework integrated into a HIL system has been proposed for real-time validation of automotive systems. Focusing on the system integration phase of the V-model, the proposed framework aims to analyze the system behavior under faults to determine the critical faults that lead to the safety violations. The corresponding behavior of the identified critical faults was then used to collect a representative dataset [69]. Despite the high coverage of the framework in injecting most of the sensor-related faults, the determination of the fault test cases was achieved manually.

In [70], Generative Adversarial Networks (GANs) and active learning are used to provide a fault injection testing technique. Causing safety failures entails the automatic generation and selection of faults. Both an active and a passive mode are available for this automated process. With a black-box testing approach that concentrates on the system’s external behavior, it is adaptive for both single and multiple fault injections.

Fiorucci et al. describe an automated method for extracting dysfunctional models from System-on-Chip designs to derive fault test cases. The objective of this procedure is to improve failure analysis for RAMS frameworks and comply with safety regulations by concentrating on bit-flip and stuck-at faults. It is a semi-automated method that combines automated extraction with systematic failure mode analysis, but it primarily targets single-event upsets or stuck-at faults. Testing combines white-box and black-box methodologies, investigating system behavior as well as internal failure modes [71].

## 4. Methodology

The Section 4 details our approach to automating fault test case generation and execution considering the drawbacks of the related works mentioned in Section 3. Specifically, we introduce a sophisticated framework employing BERT and Word2Vec to translate safety requirements into automated test cases. This section outlines the practical application of these NLP technologies. By bridging theoretical advancements with our innovative methodology, we demonstrate a significant leap forward in the field of automotive safety validations.

**Figure 2:**
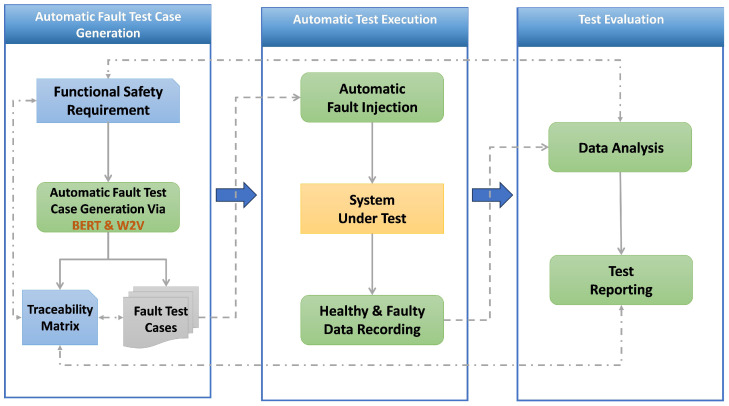
Automatic fault injection and execution approach.

Figure 2 illustrates our approach, which begins with Functional Safety Requirements, serving as the foundation for the test cases. BERT and Word2Vec are employed with a cosine similarity function to generate fault test cases relevant to the safety requirements. Fault test cases are the outcome, ready for execution. The dSPACE tools (AutomationDesk, ModelDesk, MotionDesk) facilitate Automatic Test Execution in real-time, where these test cases are applied to the System Under Test through Automatic Fault Injection depending on the black-box concept. During execution, data from both healthy and faulty states of the system are captured. Data analysis follows to evaluate the system’s performance and fault response. The process concludes with test reporting, which synthesizes the results. The entire process takes place within the HIL platform [58,59,60].

### 4.1. Automatic Fault Test Cases Generation

In this subsection, we outline the automatic generation of fault test cases. BERT [10] was fine-tuned using a specialized collection of automotive safety texts to enhance its understanding of specific terminologies within the automotive field. This included adapting the standard BERT architecture to focus on domain-specific language and customizing it for multi-label classification tasks, crucial for identifying various fault locations simultaneously.

Word2Vec [11] was employed to capture semantic similarities between different fault conditions. Trained in technical and engineering texts, Word2Vec utilized its Skip–Gram architecture [72] to predict contextual words and develop robust word embeddings. These embeddings facilitated the calculation of semantic similarities through cosine similarity measures, effectively linking safety requirements to relevant fault conditions for comprehensive test case generation.

Fault injection sites are identified by analyzing the system architecture and safety requirements. The selection of fault types is not limited by decision trees or historical data on previous faults but by selecting all faults from an extensive list to ensure thorough coverage of all possible fault test cases. This approach strictly follows ISO 26262 standards, concentrating on comprehensively testing the system’s integrity and safety without impacting its functionality.

The combined use of BERT and Word2Vec significantly improved the automation of fault test case generation and execution. Leveraging BERT’s contextual comprehension and Word2Vec’s semantic analysis capabilities allowed the system to efficiently translate detailed safety requirements into actionable test cases, supporting real-time applications in hardware-in-the-loop (HIL) simulations.

#### 4.1.1. BERT Multi-Label Classification

**Figure 3:**
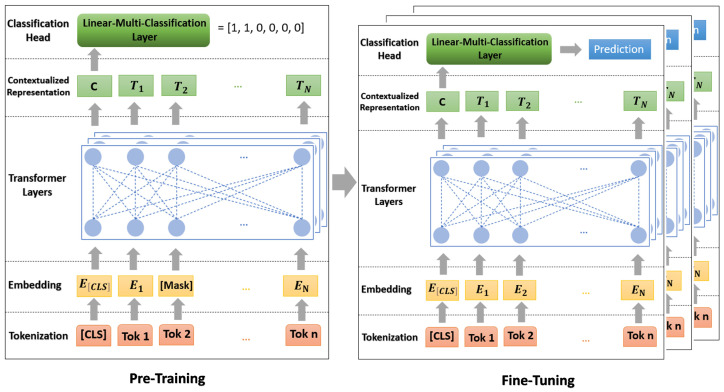
Multi-Label BERT approach.

BERT for Sequence Classification makes use of the BERT model, an advanced deep learning framework created to comprehend the context of words in text sequences. This particular variant of BERT is specifically designed to categorize texts into predefined classes. In the context of classifying functional safety requirements in vehicle systems, we adapt the BERT multi-label model by incorporating the location (the initial attribute required to inject the fault) to which the requirements are associated, as shown in Figure 3.

The main phases of the BERT approach are Tokenization, Embedding, Transformer Layers, Contextualized Representation, and Classification Head and Prediction.

1. Tokenization: Use the BERT tokenizer to tokenize the raw text (functional safety requirements) into subwords [73].
2. Embedding: Each token is transformed into an embedding following tokenization. Two different embedding types are included in this step [74]:
  (a) Token embeddings: Transform words (tokens) into vectors that represent their meaning.
  (b) Position embeddings: To give the model information about each token’s location within the sequence.
3. Transformer Layers: These layers are used for understanding the context and relationships between words in the text. The combined embeddings are then passed through BERT’s transformer layers. These layers use self-attention mechanisms to process the text non-sequentially, allowing the model to weigh the meaning of different tokens within each sequence [75].
4. Contextualized Representation: Within the Transformer levels, BERT generates contextualized token representations. These representations consider the meaning of each word as well as the context that the words surrounding it provide in a sentence [76].
5. Classification Head: “*BertForSequenceClassification*” appends a sequence classification header to the BERT model. This layer, which is a kind of dense layer, maps the number of classes in the classification task to the representation of the [CLS] token. This linear layer’s weights are trained to decipher the [CLS] token’s representation and determine the classification [77].

Multi-label classification requires fine-tuning [78,79]. A dataset unique to the multi-label task is used to train (fine-tune) the pre-trained BERT model and the recently added classification head at this stage. To better fit the features and specifications of the multi-label classification task, this procedure modifies the model’s weights.

The model finally produces a probability score independently for each label in the multi-label output [80], and a threshold value of 0.5 distinguishes between labels predicted as present (probabilities > 0.5) or absent (probabilities ≤ 0.5).

#### 4.1.2. Word2Vec and Cosine Similarity

Utilizing a computational model called Word2Vec, word embeddings—numerical representations of word features in a high-dimensional space—are created [81]. Word2Vec’s Skip–Gram [72] variant, in particular, is meant to anticipate the context of a given target word; as a result, it anticipates words that are close by in a sentence [82].

The output layer, which uses a softmax classifier, is the last layer. The softmax function compresses a K-dimensional vector of arbitrary real values into a K-dimensional probability vector where each element’s value is in the range of (0, 1) and the element sum is “1” [83].

In the training phase, the model uses backpropagation to modify its weights to make actual context words more likely and other words less likely [81]. Words that appear in comparable contexts have similar embeddings as a result of this adjustment [84].

Upon mastering the embeddings, cosine similarity will be used to calculate the semantic similarity between words. Two vectors can be compared using the cosine similarity measure, which takes into account only the direction of the vectors in vector space and ignores their magnitude [85]. According to mathematical definitions, it is as follows [86]:

(2) $$\vec{a}\cdot \vec{b}=∥\vec{a}∥∥\vec{b}∥\cos\theta .$$

(3) $$\cos\theta =\frac{\vec{a}\cdot \vec{b}}{∥\vec{a}∥∥\vec{b}∥}$$

From (2) and (3), we define (4).

(4) $$\cos\theta =\frac{\sum_{i=1}^{n}a_{i}b_{i}}{\sqrt{\sum_{i=1}^{n}u_{i}^{2}}\sqrt{\sum_{i=1}^{n}v_{i}^{2}}}$$

where *a* and *b* are two non-zero vectors, $\vec{a}\cdot \vec{b}$ is their dot-product, and $∥\vec{a}∥∥\vec{b}∥$ is their Euclidean norms.

**Figure 4:**
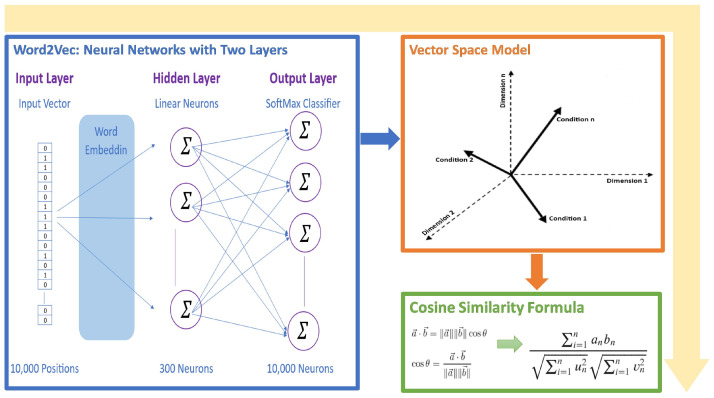
Word2Vec with cosine similarity function approach.

Leveraging a pre-trained Word2Vec model, the method translates target criteria and associated list conditions into corresponding word vectors. This translation facilitates the identification of semantically similar conditions that may differ in phrasing. By computing the cosine similarity between vectors, the method quantifies the degree of semantic alignment between each list condition and the target criterion. The condition with the highest cosine similarity score is deemed the best match, effectively harnessing Word2Vec’s contextual learning capabilities to enhance semantic search and matching processes, as shown in Figure 4.

### 4.2. Automatic Test Execution

The *dSPACE* platform offers three key tools, *AutomationDesk*, *ModelDesk*, and *MotionDesk*, each serving unique functions in automotive system testing and simulation [87].

AutomationDesk: It works seamlessly with the dSPACE tools. Python for Real-Time Testing sequences and a control-flow-based testing paradigm are required for validating control strategies, sensor fusion algorithms, and actuator responses under simulated conditions.

ModelDesk: This tool specializes in automotive simulation models (ASMs), which are open *Simulink* models that are utilized for offline and real-time simulation.

MotionDesk: This tool is intended to improve simulation realism, especially in flight and driving simulators. It can create terrain and perform “Hardware-in-the-loop” simulations.

These tools collectively enhance the capability to conduct comprehensive and realistic simulations.

### 4.3. Test Evaluation

By using dSPACE tools, we automate the execution of fault test cases including the injection into the system under test and evaluate the results of injecting the fault, which are crucial for the development and testing of automotive systems.

The evaluation is executed in two basic steps, the first is data analysis, where the system compares and analyzes the healthy and faulty data that are collected in the automatic execution of fault test cases. The second step is to report the executed test. The platform evaluates the experiments according to three different evaluation methods.

A report displays the result information that is specified in the configuration, whereas the results are saved within the project. A report can be generated for each result and appears as a child element of the result

## 5. Case Study and Implementation

Building on the theoretical underpinnings and related work discussed in previous sections, this section transitions from abstract concepts to tangible applications. This section embarks on a detailed examination of our methodology’s practical application within an **automotive steering system**, a critical component assessed at level ‘D’ based on the Automotive Safety Integrity Level (ASIL) [88]. By presenting a real-world scenario, we aim to showcase the effectiveness and adaptability of our approach, which leverages BERT and NLP technologies for the automatic generation and execution of fault test cases. This case study not only validates the concepts introduced in Section 2 and Section 3 but also showcases the operational viability and impact of our methodology on enhancing the safety validations of automotive software systems, bridging the gap between theoretical innovation and practical application.

### 5.1. System under Test

In this study, one high-fidelity Vehicle Dynamics model from [89] that simulates how a vehicle’s drivetrain and steering system behave is used. Mathematical models that simulate how an engine, transmission, differential, and steering system would react to inputs from the driver and different driving situations are also included.

A “Road Works Highway Road” scenario is used to simulate various roadwork situations on highways. The road network is designed to resemble typical highway roadwork scenarios rather than any particular real-world location [89].

**Figure 5:**
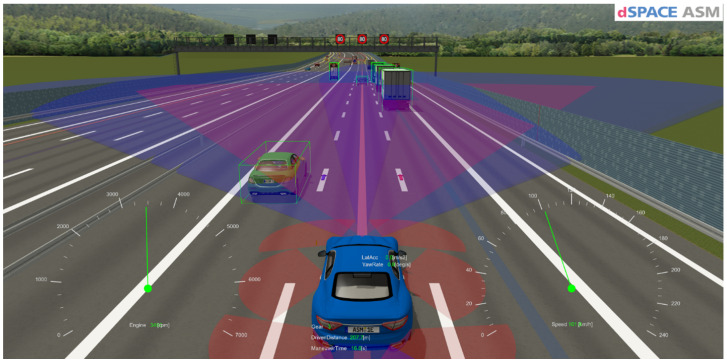
Road and traffic scenario.

The mentioned scenario was developed to mimic the movement of traffic in a highway roadwork zone. By providing a strong platform for testing and demonstrating the interactions of multiple vehicles and the effects of roadworks on highway traffic flow without the need for real map data or free trajectories on junctions, as shown in Figure 5.

### 5.2. Data Description

The data file is a dataset that encompasses a variety of aspects related to automotive steering system components. It offers an in-depth look at the intricacies of accelerator pedals, steering angles, wheel speed, yaw rate, steering torque, vehicle speed, and their interactions, specifically between the steering angle and acceleration pedal, as well as the steering angle and wheel speed. Here is a detailed description:

File Structure:

- Size: 153 kilobytes.
- Type: CSV (Comma-Separated Values).
- Lines: 1003 lines, each representing a single data point except the first row, which contains headers.

Content Overview: The dataset is structured around **eight major keywords for single-label** data and **three major keywords for multi-label** data, focusing on various components of the automotive steering system. The labels are represented as **vectors of 6 values**, accommodating both single and multi-label scenarios.

Each line in the single-label sections contain detailed information related to the following (all examples are related to the acceleration pedal):

1. **Functionality**: How components operate (e.g., converting pedal movement to electrical signals).
2. **Components and Parts**: Identifiable parts of each system (e.g., the foot pedal).
3. **Measurement and Sensors/Parameters**: Sensors involved and their parameters (e.g., position sensors).
4. **Safety and Control Systems**: Safety features and control mechanisms (e.g., drive- by-wire).
5. **Diagnostics and Troubleshooting** (e.g., diagnostic trouble code (DTC)).
6. **Vehicle Dynamics and Handling/Behavior**: How components affect vehicle performance (e.g., responsiveness, vehicle performance).
7. **Environmental Factors**: Environmental impacts (e.g., footwell, cabin environment.).
8. **Driver Behavior and Awareness/Experience**: How components influence driver experience (e.g., smooth acceleration, driving experience).

Multi-label lines focus on the following (all examples are related to the acceleration pedal):

1. **Safety and Driver Assistance**: Safety features and assistance systems (e.g., Lane Change Assist System, Lane Keeping Assist).
2. **Driver Behavior and Techniques**: The impact of systems on driver behavior (e.g., driver’s skill, driver’s control).
3. **Traffic and Road Conditions**: How systems adapt to or are influenced by traffic and road conditions (e.g., lane availability).

Label Distribution:

1. Acceleration Pedal (**Acc**): 138 lines with labels [1, 0, 0, 0, 0, 0].
2. Wheel Steering Angle (**WSA**): 160 lines with labels [0, 1, 0, 0, 0, 0].
3. Wheel Speed (**WS**): 104 lines with labels [0, 0, 1, 0, 0, 0].
4. Yaw Rate (**YR**): 107 lines with labels [0, 0, 0, 1, 0, 0].
5. Steering Torque (**ST**): 118 lines with labels [0, 0, 0, 0, 1, 0].
6. Vehicle Speed (**VS**): 102 lines with labels [0, 0, 0, 0, 0, 1].
7. Acceleration Pedal and Wheel Steering Angle (**Acc and WSA**): 142 lines with labels [1, 1, 0, 0, 0, 0].
8. Steering Angle and Wheel Speed (**WSA and WS**): 131 lines with labels [0, 1, 1, 0, 0, 0].

### 5.3. Training and Optimization

This section outlines the training and optimization process of BERT and Word2Vec models for automating fault test case generation for automotive software system validation. It emphasizes the use of (NLP) techniques to directly convert textual safety requirements into actionable fault test cases.

#### 5.3.1. BERT Model Training and Optimization

Text preparation is simplified using the “***BertTokenizer***”, which lowers all text to fit the “***bert-base-uncased***” model and divides words into smaller parts for easier processing of new words. It adds key tokens like **[CLS]** at the beginning and **[SEP]** for ends or breaks in the text, plus **[PAD]** tokens to make everything the same length. This process also creates attention masks to point out the main parts of the input, helping the model focus on the important content. The input length is capped at 512 tokens, and labels are set up in the dataset class as floating-point numbers, making everything ready for the model. The data is split, keeping 10% for testing the model on new data, and making full use of BERT’s built-in abilities for classifying different sequences.

For setting up the model, the code begins with a pre-trained “**bert-base-uncased**” BERT model suited for classifying sequences. This is loaded with a specific number of labels calculated from the dataset columns, minus one for text. It sets a standard input length of 512 tokens to fit BERT’s requirements and chooses settings like a **batch size of 16** and a **learning rate of 2 × 10^−5^** to manage how the model learns. After setup, the model is moved to the best available processing unit (**CPU or GPU**) to ensure efficient learning. This way, the pre-trained BERT model is adjusted for our specific classification task.

When it comes to optimization, the training uses the **AdamW optimizer** with a **learning rate of 2 × 10^−5^** for fine-tuning the BERT model. This optimizer is preferred because it is designed to update the model’s weights carefully to reduce loss.

Overall, the preparation, setup, and optimization processes are streamlined to adapt the BERT model for a specific sequence classification task. The tokenizer prepares the text, the model architecture is configured for the task, and the optimization is tailored for effective learning. Though it skips some advanced training techniques, the approach efficiently leverages BERT’s capabilities for this application, demonstrating how pre-trained models can be fine-tuned for specific needs with straightforward code adjustments. See Table 1.

#### 5.3.2. Word2Vec Training and Optimization

The process begins with the conversion of numbers to chars, aimed at making numerical conditions in the text more uniform for easier processing by a Word2Vec model. This function employs a ***RegEx*** pattern to identify all numbers in the text, converting them into a list of floats. If only one number is present, it is replaced with ‘a’ in the text. For texts with two, the function identifies the smallest and largest number, replacing their first occurrences with ‘a’ and ‘b’, respectively. This standardization simplifies the numerical conditions, focusing on their relational aspects rather than specific values and prepares the text for semantic analysis.

Following this preprocessing, the semantic similarity between the standardized target condition and a set of conditions from a *JSON* file is computed using a pre-trained Word2Vec model, specifically the **word2vec-google-news-300** model known for its extensive training on the Google News dataset. The conditions and the target are tokenized into words, which are then transformed into vectors using the Word2Vec’s Skip–Gram architecture to accurately capture the subtleties of word meanings in different contexts. By calculating the mean vector for the target and each condition, and then computing the cosine similarity between these vectors, the process effectively determines the semantic closeness of the conditions to the target. The condition demonstrating the highest cosine similarity to the target is identified, and its key from the *JSON* file is returned, leveraging Word2Vec’s capability to discern nuanced semantic similarities and differences within the mathematical context. This two-step process, combining numerical standardization with semantic analysis, enhances the model’s ability to identify the most relevant mathematical condition relative to the target, facilitating a deeper understanding of relational condition expressions.

#### 5.3.3. Integration between BERT and Word2Vec for Automatic Generation of Fault Test Case Attributes

**Figure 6:**
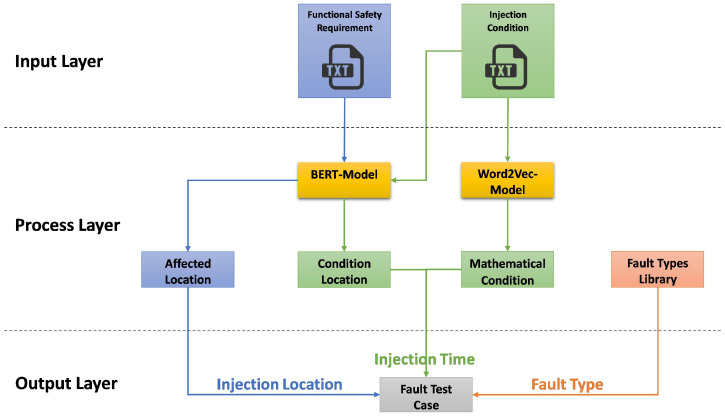
Workflow of automatic generation of fault test case attributes.

Using functional safety requirements as a starting point, the methodology—which is illustrated in Figure 6—is an automated process.

Input, Process, and Output are its three primary organizational layers. According to the figure and the description given, the following describes each layer and its elements:

1. Input Layer: This layer analyzes the text document **(FSR)**, which provides specifics about the safety tasks that the system is meant to complete. The mathematical circumstances under which faults should be injected into the system are outlined in another text document called the “**Injection Condition**”.
2. Process Layer:
  (a) BERT-Model: Based on each functional safety requirement, this model identifies one or more **affected locations** (actuators or sensor signals) by performing multi-classification. The locations where the conditions will be observed are also determined based on the relevant injection conditions.
  (b) Word2Vec and Cosine Similarity are used to recognize and match the **mathematical conditions** with a conditions database.
3. Output Layer:
  (a) The **precise location** within the system (a sensor or actuator) where the fault will be injected is known as the “Injection Location”.
  (b) As defined by the **injection condition**, the injection time (could be a specific time or an event, e.g., when the vehicle speed is 70 km/h or greater) refers to the moment at which the fault injection will occur.
  (c) Fault Type retrieved from the **Fault Type** Library.

In conclusion, the process automatically derives fault test cases from textual safety requirements and injection conditions using sophisticated NLP models such as BERT and Word2Vec. The purpose of this is to test the robustness of the system and verify that the system can adhere to the defined functional safety requirements and safely handle unforeseen conditions is the aim, by determining the appropriate location, time, and kind of fault to inject.

### 5.4. The Sequence of Automatic Execution of Fault Test Case

**Figure 7:**
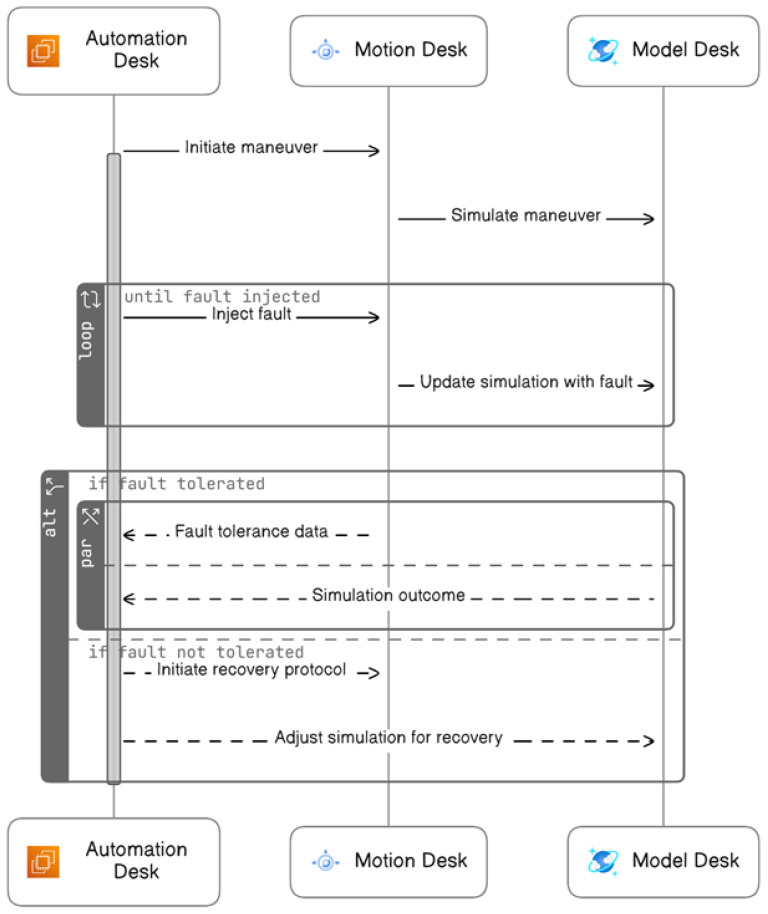
Sequence diagram of automatic execution of fault test case.

The system testing fault injection procedure is made by the *HIL* platform. This method automates the import (from the first step, “Automatic Fault Test Case Generation”) and execution of fault test cases in real-time without any human intervention using three tools *AutomationDesk*, *ModelDesk*, and *MotionDesk* as shown in Figure 7.

The following outlines an automated process for fault injection, divided into three sequential phases:

1. During the Initialization Phase, the system starts with the activation of the Motion Desk, which is the control software for simulations. This is followed by configuring and initiating the Model Desk to model the experiment environment. Finally, the capture system is configured and initiated to record data.
2. In the Fault Injection Phase, the process begins with starting maneuvers or test scenarios. Data capture commences to record the system’s behavior during these scenarios. The system then checks for the right conditions before injecting faults. Results from the fault injection are captured, and visual data representations are prepared for reporting. This phase concludes with the clearing of temporary values and stopping the test maneuvers.
3. The Cleanup Phase involves halting the data capture process and shutting down the Model Desk. Any ongoing animations are stopped, the project and experiment are formally closed, and, ultimately, the Motion Desk is shut down, completing the process.

### 5.5. Experimental Setup

This section details experiments to assess an NLP-based approach for automating fault test case generation in automotive systems. It describes two experiments: one on single fault injection and another on multiple fault injections under specific conditions. These experiments evaluate the system’s resilience to faults and its ability to maintain safety requirements. The two subsequent experiments were implemented only using a sedan vehicle equipped with an automatic transmission driving on a highway. It is worth noting that it is possible to utilize all vehicles and roads available on the dSPACE platform within this approach.

#### 5.5.1. Experiment 1: FSR for Single Fault Test Case

In the inaugural experiment of the study, we demonstrate the capabilities of an automated model to scrutinize and validate functional safety requirements within the context of a **steering system**. This system must offer unwavering feedback to the driver, reflecting the genuine steering angle and the vehicle’s dynamics.

Inputs of Experiment 1:

- **Functional Safety Requirement**: The steering system must provide consistent feedback to the driver that correlates with the actual steering angle and vehicle dynamics.
- **Condition**: No additional condition.

Steps of Experiment 1:

1. The test is initiated by providing the functional safety requirement as input.
2. The automated system, without any additional conditions, generates test cases for the steering feedback system.
3. Each test case is simulated to verify the steering feedback under normal and fault conditions.
4. Faults are injected, and the system’s response is monitored to ensure that it maintains integrity and provides accurate driver feedback.
5. Outcomes, including any deviations or failures, are logged for further analysis and reporting.

#### 5.5.2. Experiment 2: FSR for Multi-Fault Test Case

In the second experiment, the vehicle’s system must harmonize the **steering angle** with the **accelerator pedal position**, especially at higher speeds to ensure stability and prevent loss of control. The test begins under the condition that the vehicle’s speedometer indicates a speed of at least 70 km/h. At this critical speed threshold, the model initiates a multi-fault injection scenario, targeting both the steering wheel angle signal and the acceleration pedal position signal.

Inputs of Experiment 2:

- **Functional Safety Requirement**: The system must synchronize steering angle inputs with the accelerator pedal position to optimize vehicle stability and traction to prevent loss of control.
- **Condition**: The speedometer displays vehicle speed, with a minimum speed as defined being greater than or equal to 70.0 km/h.

Steps of Experiment 2:

1. The test commences by setting a predefined condition where the vehicle speedometer indicates a speed of at least 70 km/h.
2. The automated system generates multi-fault test cases targeting the steering angle and accelerator pedal signals.
3. The test injects multiple faults simultaneously under the defined speed condition to evaluate the system’s stability and control measures.
4. The system’s ability to maintain control and stability post-fault injection is assessed.
5. A comprehensive report is generated, detailing the system’s performance and its ability to mitigate risks effectively under the imposed fault conditions.

## 6. Results and Evaluations

This section presents a thorough analysis of our methodology’s effectiveness in automating fault test case generation and execution, building on the theoretical and practical insights discussed in Section 2 and Section 4. Here, we evaluate the accuracy and efficiency of our approach, utilizing BERT and Word2Vec technologies, against real-world applications and case studies. This section aims to highlight our methodology’s contributions to the evolution of automated testing in automotive software systems, showcasing its potential to enhance safety validations and set new benchmarks in the field.

### 6.1. System Behavior under Fault-Free Conditions

**Figure 8:**
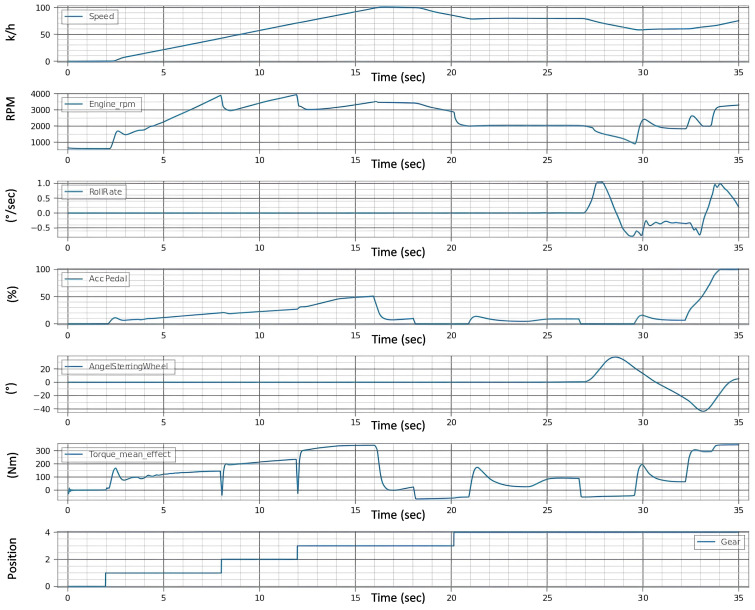
Healthy signals before fault injection.

In the experimental setup for automotive testing through fault injection in real-time, the initial phase involves conducting a baseline maneuver with the vehicle operating under normal conditions, devoid of any induced faults. This foundational stage is crucial as it establishes a reference framework, encapsulated in Figure 8, against which subsequent fault-induced scenarios are contrasted. The diagram is a visual representation of vital vehicle parameters like speed, engine RPM, and roll rate, reflecting the vehicle’s response to a dynamically changing environment with variables such as fluctuating traffic signals and variable traffic flow.

The purpose of the experiments is to inject the fault and then determine, within a tolerance of ±0.1, whether the vehicle is on the intended course. Through the analysis of signal consistency, such as steering wheel angle and speed, we can determine whether the vehicle is capable of staying on course.

A value of ±0.1 denotes the acceptable deviation in meters from the intended path on either side (y-axis) and the forward or backward (x-axis) deviation along the path. This means that the vehicle can veer up to 0.1 m from the planned route and still be considered on course.

### 6.2. System Behavior under Single Fault Conditions (Experiment 1)

This section presents findings from the first experiment focusing on the impact of a single fault injection on an automotive system’s functionality. It evaluates the system’s response to the fault. This assessment aims to determine the effectiveness of the automated fault test generation and execution methodology.

#### 6.2.1. Functional Safety Requirement of Single Fault Test Case

The first requirement stipulates that the steering system must offer consistent feedback to the driver, directly correlating with the actual steering angle and vehicle dynamics, without any additional conditions attached.

#### 6.2.2. Generation Results of Single Fault Test Case

The methodology adopted employs an automated test case generation and fault injection process that meticulously identifies the single pertinent injection site—specifically, the steering wheel angle signal. Given the absence of specific conditions in the functional safety requirement, the fault injection could be initiated at any moment during the vehicle maneuver in the experiment. Therefore, the model is programmed to begin fault injection three seconds into the maneuver, a parameter that is configurable to suit different testing scenarios. Once initiated, the fault injection process is maintained continuously until the maneuver concludes. To illustrate the methodology’s efficacy, the experiment details the injection of ‘**gain**’ in the steering wheel angle signal. The result of the suggested automatic generation methodology is shown in Table 2.

#### 6.2.3. Execution Results of Single Fault Test Case

**Figure 9:**
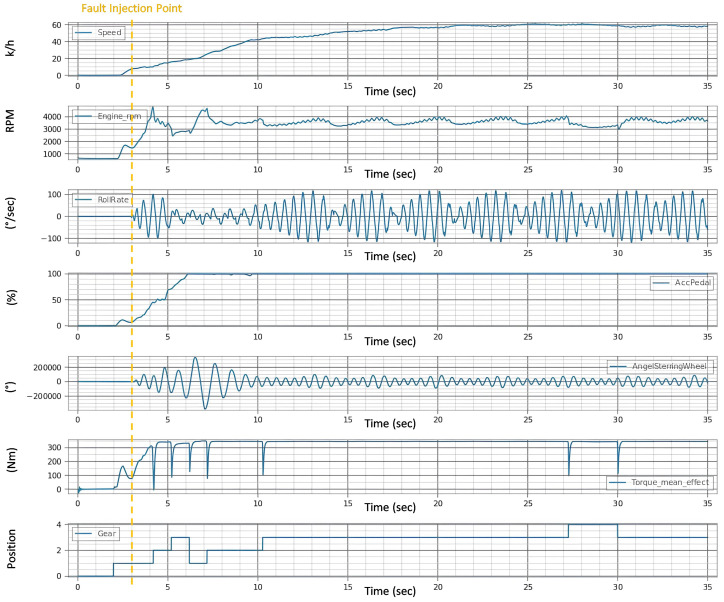
Signals after injection of single fault.

In the provided Figure 9, the test results are visually encapsulated, displaying the aftereffects of the fault on the vehicle’s operational parameters. The distortions in the signals begin after the third second, i.e., immediately after the fault is injected, which indicates the speed of the system under the test’s reaction to the faults. This graph is a pivotal piece of evidence, demonstrating the system’s behavior post-fault injection and the degree to which the fault disrupts the vehicle’s normal functioning.

#### 6.2.4. Evaluation of Single Fault Test Case

**Figure 10:**
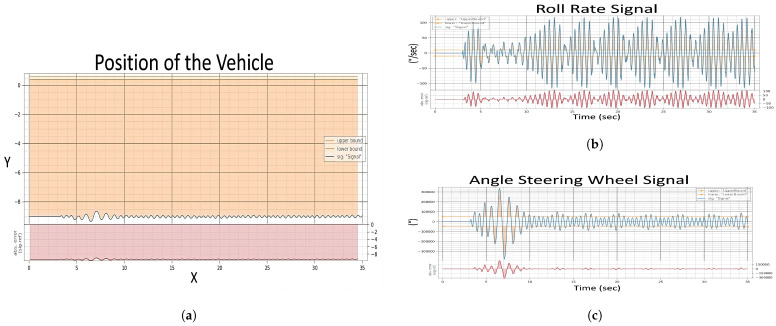
Evaluation after injection of single fault of Experiment 1, (**a**) Position of The Vehicle, (**b**) Roll Rate Evaluation, (**c**) Steering Angle Evaluation.

The evaluation of the first experiment centered on the vehicle’s ability to adhere to its intended course post-fault injection, with a strict tolerance of ±0.1 for deviation. Analysis of Figure 9 signals revealed significant instability in the fault state compared to the “Healthy Signals” Figure 8 baseline. This instability is evident in the erratic behavior of the steering wheel angle and the increased variance in the roll rate during the fault state Figure 10a, suggesting a compromised ability to maintain the course.

In the healthy state, the roll rate Figure 8 portrays minimal fluctuation, reflecting stable vehicle dynamics. However, the fault state roll rate graph exposes pronounced volatility in Figure 10b, indicating a disturbance in vehicle stability. The steering angle, when assessed, showed smooth transitions in the healthy state, which is indicative of reliable and consistent steering feedback. Conversely, in the fault state Figure 10c, the steering wheel angle’s erratic spikes breached the bounds of reasonable control, underscoring the system’s difficulty in providing the driver with accurate feedback.

### 6.3. System Behavior under Compound Fault Conditions (Experiment 2)

This section examines the outcomes of injecting multiple faults into the automotive system under a specific condition. It assesses the system’s stability and control when faced with compounded faults, specifically at higher vehicle speeds. This experiment aims to showcase the comprehensive capabilities of the proposed automated testing approach in identifying complex safety risks.

#### 6.3.1. Functional Safety Requirement of Multi-Fault Test Case

The second requirement emphasizes the system’s ability to synchronize steering angle inputs with the accelerator pedal position, especially when the vehicle speed is 70.0 km/h or higher, as indicated by the speedometer.

#### 6.3.2. Generation Results of Multi-Fault Test Case

The model responded to this condition exactly when the speed reached 70.0 km/h. For this test, nine different faults are introduced in pairs, resulting in 81 unique fault cases. When two faults are simultaneously injected into separate components of a system under test, it is referred to as Multi-Faults. Experience [69] shows that if there is a relationship between the features of these two components, the behavior of the system will differ from what would be observed if each fault were injected individually. To illustrate the methodology’s efficacy, the experiment details the injection of ‘**noise**’ in the steering wheel angle signal and ‘**drift**’ in the acceleration pedal signal. The result of the suggested methodology is shown in Table 3.

#### 6.3.3. Execution Results of Multi-Fault Test Case

**Figure 11:**
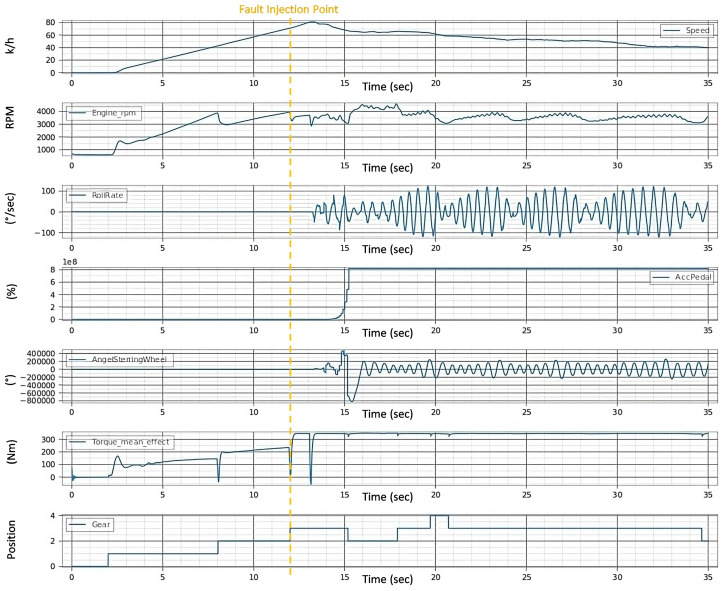
Signals after injection of multi-fault.

Figure 11 serves as a testament to this process, illustrating the effects of concurrent noise faults in the steering signal and drift faults in the acceleration pedal. The model injects these faults in real time. The distortions in the signals begin when the vehicle speed reaches 70.0 km/h (approx. 12 s later), i.e., immediately after the fault is injected, which indicates the speed of the system under the test’s reaction to the faults. This experiment not only tests the system’s adherence to safety requirements under compounded fault conditions but also demonstrates the precision and adaptability of the automated testing approach in a dynamic driving environment.

#### 6.3.4. Evaluation of Multi-Fault Test Case

In Experiment 2, the evaluation is predicated on the vehicle’s system’s ability to align steering angle inputs with the accelerator pedal position at a critical speed threshold to enhance stability and prevent control loss. Figure 11 illustrates the system’s behavior under a dual fault condition—a noise fault in the steering wheel angle signal and a drift fault in the acceleration pedal signal—when the vehicle’s speedometer reads speeds exceeding 70 km/h.

**Figure 12:**
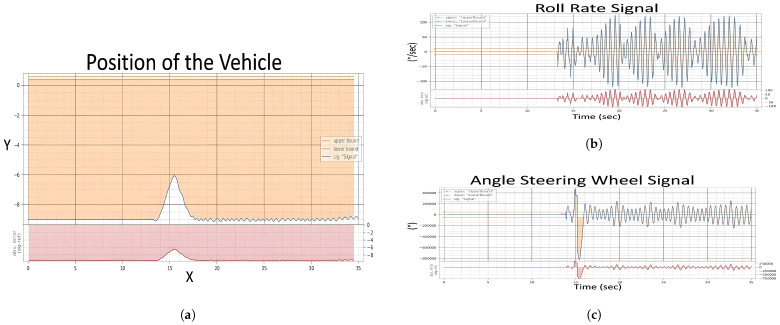
Evaluation of Experiment 2, (**a**) Position of The Vehicle, (**b**) Roll Rate Evaluation, (**c**) Steering Angle Evaluation.

Figure 11 shows how these faults affect vehicle dynamics, with the roll rate Figure 12b indicating increased volatility, and the steering angle signal Figure 12c exhibiting erratic deviations beyond expected boundaries. The vehicle position Figure 12a further demonstrates the challenges in maintaining the intended trajectory, highlighting the system’s struggle to synchronize steering and acceleration inputs under fault conditions.

In both experiments of fault injections (single and multi-fault) in Figure 9 and Figure 11, the following has been observed:

- The observed increases in speed and torque mean effort suggest heightened energy consumption, potentially reducing fuel efficiency.
- A stable but slightly variable engine RPM indicates that the engine is not always operating in inefficient ranges.
- Oscillations in the roll rate are indicative of stability control systems consuming additional energy to maintain vehicle balance.
- Additionally, frequent steering corrections and erratic gear shifts, especially noted in the multi-fault scenario Figure 11, may lead to dynamic inefficiencies and less optimal engine performance, further affecting energy use and fuel economy.

These changes in operational parameters, following fault injections, suggest a complex interplay of vehicle systems that could negatively impact overall performance and efficiency.

### 6.4. BERT Model Evaluation

#### 6.4.1. BERT Model Training Evaluation

Table 4 appears to be an evaluation output for a BERT (Bidirectional Encoder Representations from Transformers) model across different epochs during its training phase. Each epoch represents a full pass of the training dataset through the neural network. The table includes metrics such as Accuracy, F1 Score, Loss, and Hamming Loss, which are used to evaluate the performance of a machine learning model.

**Figure 13:**
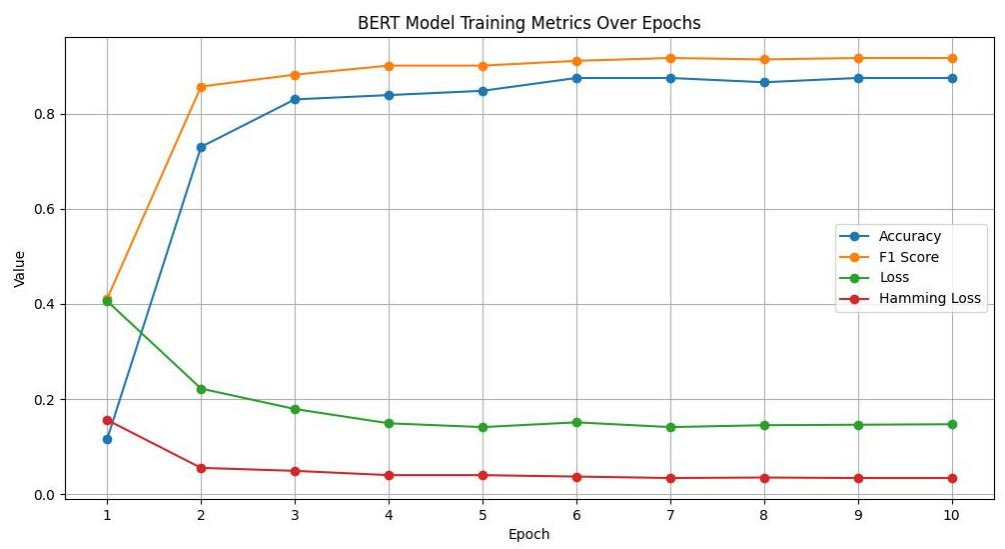
BERT model training metrics over epochs.

Figure 13 compares the BERT model’s accuracy and loss throughout ten epochs. The correlation between accuracy and loss is inverse, as predicted. The model is learning and getting better at making predictions over time when accuracy rises and loss falls. Accuracy and loss metrics start to stabilize after some initial fluctuations. This means that the model is approaching convergence and that there is a point at which more training yields less and less benefit. Beyond the sixth epoch, accuracy peaked at 0.875, and the loss likewise exhibits very little variation. Given the present setup and dataset, this suggests that the model is operating at its peak efficiency.

The F1 score and accuracy are compared throughout the BERT model training epochs. Indeed, early epochs show rapid improvements in both the F1 score and accuracy, suggesting that the model is effectively learning from the training set. Given the current dataset and model architecture, the model may have reached its peak performance because both metrics plateaued after the sixth epoch. The model may be better at identifying true positives and true negatives than it is at striking a balance between precision and recall if the F1 score is typically lower than the accuracy. This might occur if there is a class imbalance in the data or if the model produces conservative predictions.

The accuracy and F1 score were compared across the epochs with the Hamming loss. All three metrics plateaued around the sixth epoch, which implies that the model might not benefit from additional training using the available data and configuration. The instance-wise label prediction errors decrease as the model’s overall performance improves, which is a desirable outcome in multi-label classification problems, according to the inverse relationship between the Hamming loss and the other two metrics (F1 score and accuracy).

#### 6.4.2. BERT Model Testing Evaluation

In this part we delve into the BERT model’s performance evaluation, testing it against a dataset comprising 84 functional safety requirements (FSRs). With 14 FSRs dedicated to each single-label class—Acceleration Pedal (Acc), Wheel Steering Angle (WSA), Wheel Speed (WS), Yaw Rate (YR), Steering Torque (ST), and Vehicle Speed (VS)—and an additional 28 FSRs for multi-label classes (14 each for ACC and WSA and WSA and WS). The model showed very good accuracy (91.07%) when handling it.

**Figure 14:**
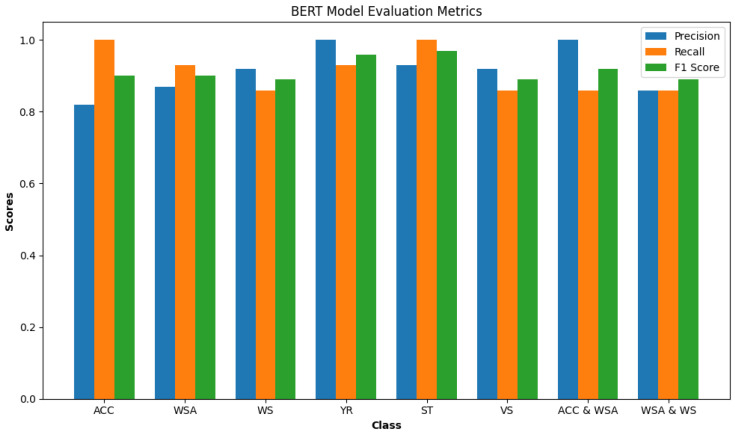
BERT model metrics by class.

In Figure 14, we see the evaluation metrics for a BERT model for each class on a test dataset. The precision of the model is 91.5%. The recall (sensitivity) is 91.25%. The F1 score is 91.5%.

Precision: Every item predicted as Yaw Rate (YR) or Acceleration Pedal and Wheel Steering Angle (Acc and WSA) was indeed correct (both at 1.00). The lowest precision is for the Acceleration Pedal (Acc) class (0.82), indicating some false positives.

Recall: Acceleration (Acc), Steering Torque (ST), and Vehicle Speed (VS) classes have perfect recall (1.00). Wheel Speed (WS) and Wheel Steering Angle and Wheel Speed (WSA and WS) have the lowest recall (0.86), suggesting that the model missed some true instances of these classes.

F1 Score: The Steering Torque (ST) class has the highest F1 score (0.97), indicating an excellent balance between precision and recall. Wheel Speed (WS) and Wheel Steering Angle and Wheel Speed (WSA and WS) classes have the lowest F1 score (0.89).

**Figure 15:**
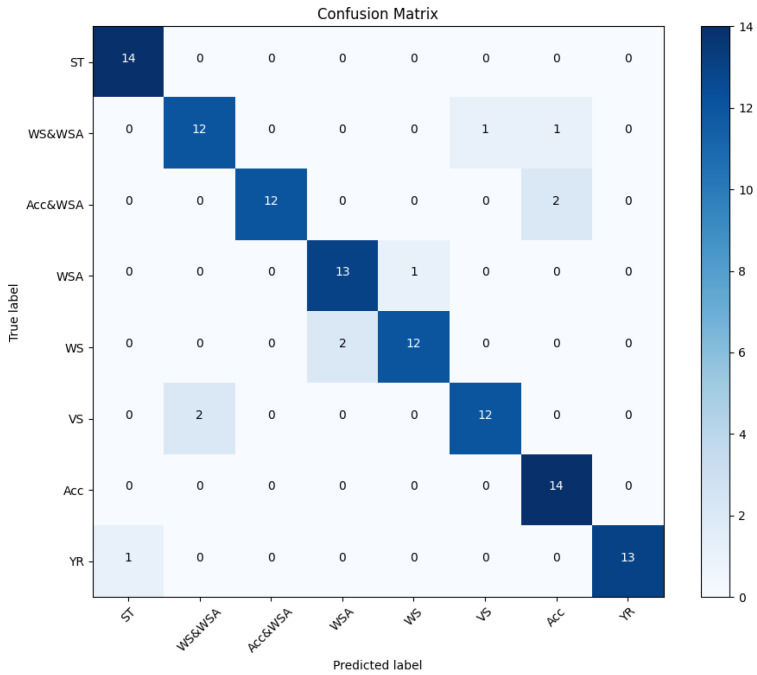
BERT testing confusion matrix.

From the confusion matrix Figure 15 conclude that imbalance does not affect the model evaluation, because there is a uniform distribution of class across all classes in the test set. Vehicle Speed (VS) has been misclassified as Acceleration Pedal (ACC) twice, because of the similarity in the features between these classes that the model is picking up on. The precision for the Acceleration Pedal (Acc) is notably lower compared to other classes, which could indicate that the model is overly optimistic in predicting this class (because the strong relationship between the accelerator pedal and other classes such as vehicle speed and wheel speed) and could be improved by further training or adjusting the classification threshold. The classes Wheel Speed (WS) and Wheel Steering Angle and Wheel Speed (WSA and WS) have the same recall and F1 score, suggesting that the model might be confusing these two classes with each other or with other similar classes. The BERT model appears to perform very well on this test set, with high precision, recall, and F1 scores for almost all classes. The balance between precision and recall is indicated by the F1 scores, which are generally high. Some areas could be improved, such as

- Working on distinguishing Acceleration Pedal (Acc) features more clearly to increase the precision and reduce the false positives.
- Analyzing the misclassifications to understand if commonalities are causing the errors to address the confusion between (WS), (WSA and WS), and possibly other classes.

### 6.5. Word2Vec Model Testing Evaluation

In this part, we present a detailed evaluation of the Word2Vec model’s testing performance, where 50 conditions were analyzed to determine their similarity to our dataset. The dataset encompasses 10 distinct conditions, each subjected to five rigorous tests to ascertain the most analogous condition from the test dataset to our own.

**Figure 16:**
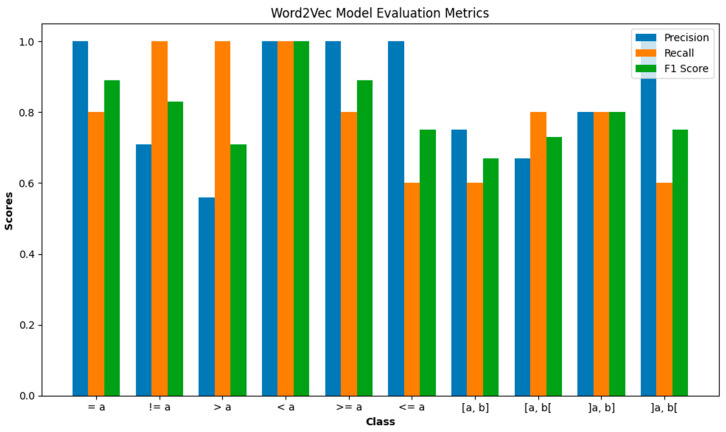
Word2Vec model evaluation metrics.

In Figure 16, we see the evaluation metrics for a Word2Vec and Cosine Similarity Function model for each class on a test dataset. The precision of the model is 84.9%. The recall (sensitivity) is 80%. The F1 score is 80.2%.

Precision: The model performs perfectly in identifying (<=a), (<a), and (>=a) conditions with a precision of 1.00. However, it struggles with (>a) and [a, b[, showing a precision of 0.56 and 0.67, respectively, indicating a higher rate of false positives.

Recall: The (>a), (<a), and (!=a) conditions are perfectly identified with a recall of 1.00. The lowest recall at 0.60, indicating that the model missed 40% of the relevant instances.

F1 Score: High scores for (=a) and (>=a) indicate a good balance between precision and recall. The conditions (>a) and [a, b] have lower F1 scores, indicating an area for improvement.

**Figure 17:**
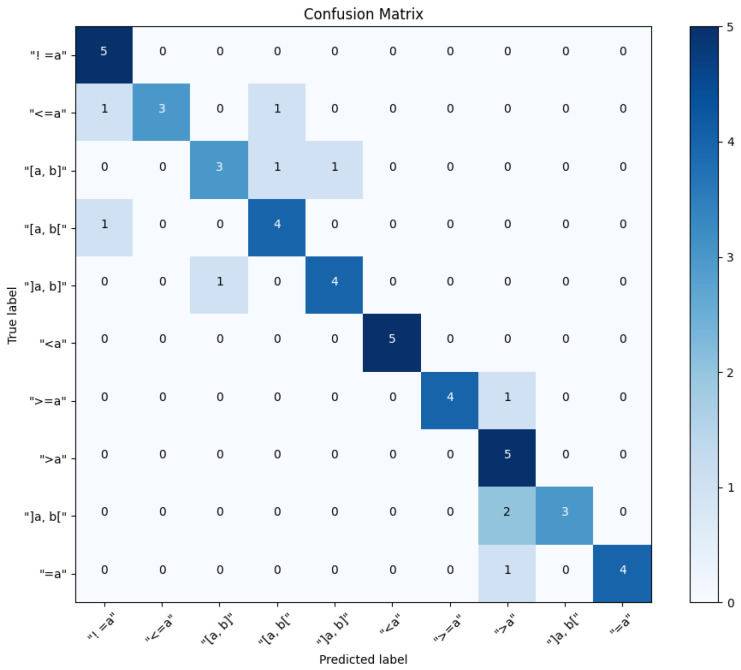
Word2Vec testing confusion matrix.

Based on the confusion matrix in Figure 17. All classes have been tested equally (5 times each), totaling 50 tests. This balanced testing allows for an even comparison of performance metrics across classes. The model is most confident and accurate (86.67%) in identifying conditions with one comparing value (a) and less accurate (65%) in identifying conditions with closed intervals value (a, b). The comparison between values like (!=a) and (=a) shows a contrast in recall despite both dealing with equality.

In Table 5, the steps of manual generation and execution of fault test cases are compared with our automatic approach.

## 7. Conclusions and Discussion

This article presents a novel approach that automates the generation and execution of fault test cases for automotive safety systems by leveraging real-time fault injection. By integrating natural language processing technologies, specifically BERT and Word2Vec. The methodology demonstrated high efficacy, with the BERT model achieving 91.07% accuracy. The precision of the model is 91.5%. The recall (sensitivity) is 91.25%. The F1 score is 91.5%. The Word2Vec model showed a precision of 84.9% and an F1 score of 80.2%, effectively identifying semantically similar conditions for fault injection. We have devised a system that can transform functional safety requirements directly into fault test cases. These cases are then executed on a Hardware-in-the-Loop (HIL) platform, demonstrating the practical application of our approach.

Both experiments yielded definitive outcomes, confirming the efficacy of our approach. The objective was to validate the potential of NLP technologies in automating critical test processes, thus enhancing the efficiency and effectiveness of fault injection in safety validations in the automotive domain. The successful conclusion of these experiments underscores the viability of NLP in improving the precision and speed of fault injection approach assessments.

The successful application of NLP techniques and machine learning models in this study opens the door to a host of possibilities for future works:

- Explore the integration of advanced Transformer-based models like RoBERTa and GPT, which may offer enhanced understanding and generation capabilities, potentially increasing the precision of test case generation in complex automotive software systems.
- The methodology demonstrated here holds significant potential for adaptation to other safety-critical domains such as aerospace and medical devices, where rigorous testing regimes are similarly essential to ensure operational safety under diverse conditions.
- Further research could involve utilizing the established methodology in a simulated real-world environment where actual users drive the vehicle through a simulator.
- Additionally, expanding the research to cover more extensive error domains would help in identifying and rectifying potential shortcomings.

## Figures and Tables

**Table 1:** Preparation, setup, and optimization processes.

Phase	Parameter	Value
Text Preparation	Model Name	bert-base-uncased
	Maximum input length	512
	Special Tokens	[CLS], [SEP], [PAD]
Model Architechture Setup	Batch Size	16
	Learning Rate	2 × 10^−5^
	Device	CPU or GPU
	Number of Labels	len(df.columns) − 1
Optimization	Optimizer	AdamW

**Table 2:** Generation output of Experiment 1.

Injection Location	Injection Time	Fault Type	Single/Multi Fault
Steering wheel angle signal	On specific time	List of nine faults	Single fault injection

**Table 3:** Generation output of Experiment 2.

Injection Location	Injection Time	Fault Type	Single/Multi Fault
Steering wheel angle and acceleration pedal signals	Vehicle speed ≥ 70 km/h.	List of nine faults in pairs	Multi-fault injection

**Table 4:** BERT fine-tuning epochs.

	Epoch 01	Epoch 02	Epoch 03	Epoch 04	Epoch 05	Epoch 06	Epoch 07	Epoch 08	Epoch 09	Epoch 10
Accuracy	0.116	0.730	0.830	0.839	0.848	0.875	0.875	0.866	0.875	0.875
F1 Score	0.411	0.857	0.882	0.901	0.901	0.911	0.917	0.914	0.917	0.917
Loss	0.406	0.222	0.179	0.149	0.141	0.151	0.141	0.145	0.146	0.147
Hamming Loss	0.156	0.0553	0.049	0.040	0.040	0.037	0.034	0.035	0.034	0.034

**Table 5:** Comparison between manual and automatic approaches.

Step	Explanation	Manual Approach	Automatic Approach
Supervisor	Supervising the experiment	Expert human	System
FSR analysis	Reading, understanding, and deriving fault test case attributes (location(s), injection time, fault type)	4 min	36 s
Experiment setup	start relevant dSPACE tools ^1^ then upload vehicle and road scenario	3 min	90 s
Start the Experiment	Start the maneuver and choose signals for recording	2 min	35 s
Execution of fault Test Case attributes	Location(s) ^2^, time ^3^, fault type	30 s	
Stop the Experiment	Stop the maneuver and signal recording	5 s	
Reporting	System pass/fail and evaluations	10 min	2 s

^1^ The relevant dSPACE tools for the manual approach are ControlDesk, ModelDesk, and MotionDesk, but for the automatic approach, they are AutomationDesk, ModelDesk, and MotionDesk. ^2^ In the manual approach, multi-fault case the user should inject the faults in two different locations at the same time and it is impossible, which affects the experiment’s results. ^3^ Human users cannot inject the fault at the right time especially when the system’s under-test behavior changes rapidly, which could lead to repeating the experiment.

## Data Availability

Data available on request due to restrictions.

## Abbreviations

The following abbreviations are used in this manuscript:

ASM	Automotive Simulation Models
BERT	Bidirectional Encoder Representations from Transformers
FI	Fault Injection
FSR	Functional Safety Requirement
HIL	Hardware-In-the-Loop Simulation
NLP	Natural Language Processing
SUT	System Under Test
